# Deproteinization as a Rapid Method of Saliva Purification for the Determination of Carbamazepine and Carbamazepine-10,11 Epoxide

**DOI:** 10.3390/jcm9040915

**Published:** 2020-03-27

**Authors:** Ewelina Dziurkowska, Marek Wesolowski

**Affiliations:** Department of Analytical Chemistry, Medical University of Gdansk, Gen. J. Hallera 107, 80-416 Gdansk, Poland; marek.wesolowski@gumed.edu.pl

**Keywords:** saliva, deproteinization, carbamazepine, carbamazepine-10,11 epoxide

## Abstract

Saliva is a valuable diagnostic material that, in some cases, may replace blood. However, because of its different composition, its use requires the development of new, or the modification of existing, extraction procedures. Therefore, the aim of the study was to develop a method of saliva purification that would enable the determination of carbamazepine and its metabolite, carbamazepine-10,11 epoxide. When comparing two methods of sample purification (Solid Phase Extration (SPE) and deproteinization), it was found that the second method yielded more favorable results. A 1% formic acid solution in acetonitrile was used for extraction. The samples were shaken and centrifuged, and the supernatant obtained was evaporated and dissolved in a mobile phase, then chromatographically analyzed. The developed method was validated by determining its linearity in the range of 10–5000 ng/mL for both analytes. Intra- and inter-day precision did not exceed 14%. In order to check the usefulness of the method, both analytes were determined in the saliva samples from 20 patients treated with carbamazepine. The content of both analytes was detected and determined in all of the tested samples of saliva. It was found that the method developed is rapid, sensitive, reliable, and can be used to monitor the concentration of carbamazepine and metabolite in patients’ saliva.

## 1. Introduction

For many years, saliva has been the subject of research aimed at making use of it as an easily accessible diagnostic material. Its collection does not require the presence of trained staff, and the patient can take the sample at home and deliver it to the laboratory. Saliva sampling does not cause any additional stress, which is extremely important for determining the levels of substances such as cortisol. Unlike blood sampling, it is also much less stressful to children. Monitoring the level of active substances in saliva can be the method of choice for the elderly, in whom blood collection is hindered by the fragility of veins. Moreover, in the case of persons who have committed a crime, taking a sample of saliva prevents one from falsifying or submitting for analysis a sample received from another person, which may be the case when urine samples are taken [1,2,3].

Despite its many advantages, for many years, saliva has not been the basic biological material for the analyses of active substances, because very often, the concentration of the monitored substances in saliva is lower than in blood. Therefore, the determination of analytes in saliva requires a more sensitive analytical method or a larger sample volume. Moreover, the composition of saliva is different from that of blood, which necessitates the creation of new methods or the modification of already existing procedures for blood in order to use them for saliva analysis [1,2].

**Figure 1:**
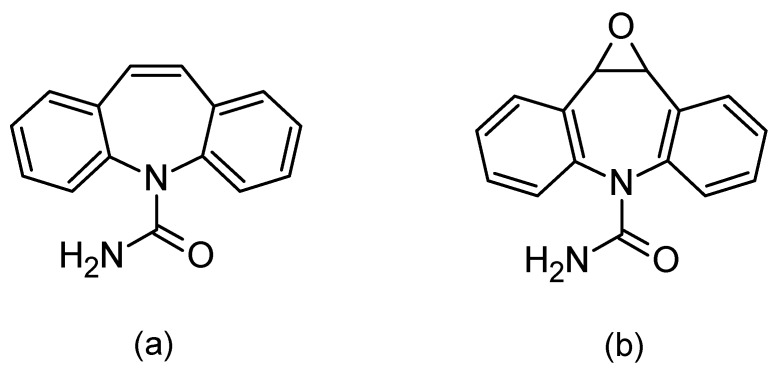
Chemical formulas of carbamazepine (**a**) and its active metabolite, carbamazepine-10,11 epoxide (**b**).

When using saliva to monitor the concentration of drugs in the body, special attention should be paid to the correlation between the concentration of the test substance in the blood and saliva. Such a correlation allows for using saliva as a diagnostic material. In the absence of a correlation, saliva may provide a convenient matrix for abuse control, e.g., abuse of psychoactive agents. One of the drugs for which the correlation between differences in the blood and saliva concentration has been confirmed is carbamazepine (Figure 1a) [2,3,4,5,6,7,8,9].

Carbamazepine was discovered in 1953 and introduced into treatments in 1962 as an antiepileptic, as well as being used for treating neuropathic pain. Moreover, in combination with neuroleptics, it is used to treat schizophrenia and as a mood stabilizer in bipolar disorder [8,10,11]. The mechanism of carbamazepine is to block the sodium channels. Its half-life after a single administration is 36 h, whereas after repeated administration, due to strong hepatic enzyme induction, the half-life is shortened and ranges between 16 and 24 h. It is metabolized by isoenzyme from the group of cytochrome P450CYP3A4 to active epoxide (carbamazepine-10,11 epoxide; Figure 1b), and is excreted in the urine (72%) [10,11,12].

Carbamazepine may affect the metabolism of other drugs metabolized by this isoenzyme, e.g., reducing the concentrations of clozapine, quetiapine, and aripiprazole. When used concurrently with lithium, carbamazepine may intensify lithium’s neurotoxic effects. Because of the nonlinear pharmacokinetics of carbamazepine and its active metabolite, as well as the numerous adverse effects and high individual variability in the dose-to-plasma concentration resulting from numerous drug interactions, it is advisable to monitor these analytes and personalize the dose-to-plasma concentration. Carbamazepine and carbamazepine epoxide concentrations in the body should be monitored, particularly in children and adults, when other drugs metabolized by isoenzyme CYP3A4 are administered simultaneously [13]. In this case, monitoring can be carried out using saliva samples.

Saliva sampling for the analysis of the carbamazepine content is most often taken following stimulating its secretion by chewing Parafilm [3,9,14] or eating citric acid [15,16,17,18]. Saliva samples can also be collected in a tube without prior stimulation [8,9,10,11,12,13,14,15,16,17,18,19,20] or on cotton swabs specially designed for this purpose, which are placed in the mouth to soak up the saliva [21,22,23]. After removing the swab from the mouth, it is placed in a tube and is centrifuged.

The most common method of carbamazepine isolation from saliva samples is liquid–liquid extraction [8,14,16,24,25,26]. Saliva samples are also purified using a modified liquid–liquid extraction, cloude-point extraction [26], solid phase extraction [22,23], and saliva deproteinization [15,20].

Carbamazepine in saliva can be quantified by an enzymatic method (FPIA—fluorescence polarization immunoassay) [7,19], as well as by separation techniques, gas chromatography [2,16,27], and liquid chromatography coupled with UV detection [3,8,9,14,15,17,18,20,21,22,23,24,25,26].

Taking into account the potential benefits that may arise from the use of saliva as a diagnostic material, the aim of this study was to develop extraction conditions and to validate a method for the determination of carbamazepine and its active metabolite in saliva samples. The intention was to come up with an easy, rapid, and accurate method of saliva sample purification, and the volume of sample needed to determine the concentration of analytes. The developed method requires only 200 µL of saliva, which is significant, as the determinations using saliva are particularly recommended for children from whom blood collection may be very stressful.

The significance and newness in the developed method in relation to the existing ones is based on the fact that the developed method allows for the determination of carbamazepine and its metabolite in a small saliva sample collected with salivette, which is used to help with secretion of an appropriate amount of saliva. Moreover, our study revealed that the Solide Phase Extraction (SPE) Phree columns designed for analysing small blood volumes cannot be used for saliva purification. However, the use of deproteinization was the most effective for this purpose.

## 2. Experimental Section

### 2.1. Instruments

The chromatographic analysis was performed with a Nexer XR UHPLC (Ultra High Performance Liquid Chromatography) liquid chromatograph (Schimadzu, Kyoto, Japan), equipped with a SPD-M30A UV-Vis detector with a diode array and a highly sensitive measuring cell SPD-M30A (85 mm), CBM-20 Alite control system, LC-30AD pump, SIL-30AC autosampler, and CTO-20AC thermostat (Shimadzu, Kyoto, Japan). The compounds were separated using a Luna Omega 1.6 µm Polar C18 (LC Column 50 × 2.1 mm ID) with pre-columns (UHPLC Fully Porous Polar C18, 2.1 mm ID). The compounds were eluted from the column at 35 °C with a mobile phase consisting of water with added formic acid and triethylamine (TEA) (solvent A, pH 4.2) and acetonitrile (solvent B) at a flow of 1 mL/min. The composition of the mobile phase and its changes during the analysis are presented in Table 1.

### 2.2. Chemicals and Solvents

Acetonitrile, methanol, and formic acid were obtained from POCh (Gliwice, Poland), and TEA was supplied by Sigma-Aldrich (St. Louis, MO, USA). All of the reagents had HPLC super grade purity. The deionized water was purified by Ultra-Toc/UV, Hydrolab (Straszyn, Poland). Standards of carbamazepine came from Polpharma (Starogard Gdański, Poland). Carbamazepine-10,11 epoxide solution (1 mg/mL) was obtained from Sigma-Aldrich (St. Louis, MO, USA). The internal standard (IS), chlordiazepoxide, was purchased from Polfa Tarchomin (Warsaw, Poland).

Standard solutions of carbamazepine and chlordiazepoxide (1 mg/mL) were prepared by weighing 10 mg of the substance and dissolving in 10 mL of methanol. Working solutions of carbamazepine, carbamazepine-10,11 epoxide, and chlordiazepoxide were prepared by diluting the standard solutions with methanol. All of the working solutions were stored at −21 °C.

### 2.3. Saliva Sampling

Saliva samples for preliminary examination and method validation were taken using Salivettes (Sarstedt, Nümbrecht, Germany). Healthy volunteers were required to refrain from eating and drinking liquids 30 min before sampling, and were asked to wash their mouth with water 10 min before placing a Salivette in their mouths and chewing it for about 2 min. The swab was then placed back in the tube and centrifuged for 5 min at 8000 rpm. Centrifuged samples were frozen and stored at −20 °C until analysis.

### 2.4. Saliva Treatment

The saliva samples were thawed, centrifuged, and then 200 μL of test material was collected and placed in plastic tubes. A 1% formic acid solution in acetonitrile (600 µL) was added to each of them, mixed with a vortex, shaken (laboratory shaker 358 S, Elpin, Poland) for about 10 min, and centrifuged for 5 min at 8000 rpm. The supernatant was transferred to glass tubes and evaporated. The dry residue was dissolved in 100 μL of an acetonitrile/water mixture with formic acid (1:10), and 10 μL was injected into the chromatographic column.

### 2.5. Method Validation

#### 2.5.1. Linearity

The linearity of the method was determined by performing four calibration curves on four consecutive days. The curves were prepared by adding appropriate volumes of working solutions to 200 μL of saliva, so that the final IS concentration was 500 ng/mL, while the analytes were 10, 50, 100, 500, 1000, 3000, and 5000 ng/mL, respectively. The acceptance criteria for the calibration curve included a coefficient of determination (R2) ≥0.99 and residuals ≤15% at each concentration level.

#### 2.5.2. Selectivity

The selectivity of the method was determined by analyzing 10 blank saliva samples obtained from 10 healthy volunteers. The study was conducted to detect interference resulting from the presence of endogenous compounds. Their absence allows for the method to be considered selective.

#### 2.5.3. Precision and Accuracy

Intra- and inter-day precision and accuracy were tested at three concentrations (30 ng/mL—low quality control (QC); 2000 ng/mL—medium QC; 3750 ng/mL—high QC). For intra-day precision, five samples of each concentration were analyzed on one day. In the case of inter-day precision, one series of each concentration was analyzed over four consecutive days (*n* = 20). The precision of the method was expressed by the coefficient of variation (%CV), taking ≤ 15% as an acceptable value. The accuracy was expressed as the percentage of nominal concentration, with the threshold within 85–115% of the target concentration.

#### 2.5.4. Limits of Quantification

The limit of quantification (LOQ) for the tested concentrations was repeated five times, and its value was considered to be the lowest concentration of analytes for which the signal was ten times higher than the noise, the precision of CV determination was <20%, while the recovery of the analyte was ± 20%.

#### 2.5.5. Absolute Recovery and Extraction Recovery

The absolute recovery and extraction recovery were determined for two concentrations of analytes (30 ng/mL—QC low; 3750 ng/mL—high QC). Six saliva samples were prepared for each concentration by adding to each sample an appropriate volume of working solution of the analyte and IS. The samples were analyzed according to the procedures described in Section 2.1 and Section 2.4.

In order to determine the absolute recovery, the peak areas of the extracted analytes were compared with the peak areas obtained from the analysis of six neat standards of each concentration. The extraction recovery was determined by comparing the surface areas of the peaks of the analytes subjected to the extraction process, with the peak areas of each concentration of the analytes obtained by analyzing blank saliva samples, which were loaded with the solutions of the analytes after extraction. The average of six neat standards of each concentration was assumed to be 100%, while an acceptable result was considered to be the one for which the analyte’s value exceeded 50%.

#### 2.5.6. Stability

The stability of the analytes was tested for three concentrations (low QC, medium QC, and high QC). The stability of the analytes was determined both in the matrix during storage at 8 °C and in a freeze–thaw test, during which spiked saliva samples were stored at −21 °C. For each concentration of carbamazepine and metabolite, three 800 μL saliva samples were prepared in plastic tubes, spiked with an appropriate volume of analytes, and mixed. Then, 200 μL of saliva was taken from each sample, IS added, extracted, and chromatographed according to the procedures described in Section 2.4 and Section 2.5. The remainder of the sample was placed back in the refrigerator or frozen and analyzed in the following days.

The stability of the analytes was also determined during their storage in an autosampler at 15 °C for 72 h. For this purpose, five samples of each concentration were extracted and chromatographed. After 72 h, the samples were then chromatographed again alongside a freshly prepared calibration curve.

The compounds whose concentration decreased by less than 15% under the given storage conditions were considered to be stable.

### 2.6. Clinical Application

The usefulness of the method was determined by examining the saliva samples of 20 patients treated with carbamazepine preparations. They were collected from patients of the Nervous and Mentally Ill Hospital in Starogard Gdański (Poland). The study protocol was approved by the ethical committee of the Medical University of Gdansk, Poland (NKBBN/139/2016). Saliva was taken using Salivettes about 2 h after the administration of carbamazepine. The Salivette was placed in a test tube and centrifuged, and the obtained filtrate was frozen at −21 °C and stored until analysis. To perform the analyses, the samples were thawed, centrifuged, and 200 μL of liquid taken. Then, 50 μL of IS solution (10 μg/mL) was added and the procedure described in Section 2.4 was followed.

## 3. Results

### 3.1. Chromatographic Analysis

**Figure 2:**
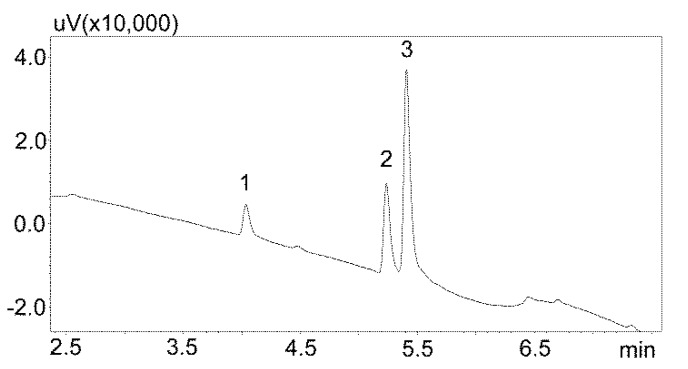
Chromatogram of the standard solutions obtained by optimized UHPLC. The compounds were separated using a Luna Omega 1.6 µm Polar C18 column and a mobile phase consisting of water with formic acid and (solvent A) and acetonitrile (solvent B) at a flow of 1 mL/min. 1—carbamzepine-10,11 epoxide; 2—carbamazepine; 3—IS (internal standard: chlordiazepoxide).

The first stage of chromatographic separation optimization included the selection of the composition of the mobile phase, its flow rate, and the internal standard. The composition of the mobile phase and its appropriate flow ensured the optimal separation of the analyzed substances from other components of the matrix. Chromatographic analysis lasted 15 min and peak detection was performed at 240 nm. Chlordiazepoxide was used as the IS. This benzodiazepine derivative is not currently used in medicine, and therefore may not be present in patients’ saliva. Furthermore, chlordiazepoxide shows good UV absorption at 240 nm. The chromatogram obtained by analyzing standard solutions using optimized chromatographic analysis conditions is shown in Figure 2.

### 3.2. Method Development

#### 3.2.1. Extraction with Phree Columns

**Figure 3:**
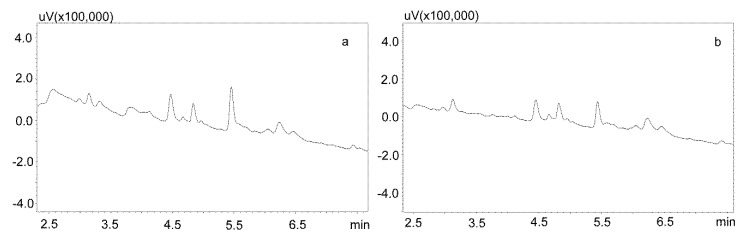
Chromatograms of blank saliva extracted with Phree columns: (**a**) extracted with 1% formic acid in methanol; (**b**) extracted with 1% formic acid in acetonitrile. The separation was carried out using a Luna Omega 1.6 µm Polar C18 column and a mobile phase consisting of water with formic acid (solvent A) and acetonitrile (solvent B) at a flow of 1 mL/min.

Phree phospholipid removal plates (Phenomenex, Torrance, CA, USA) were used to optimize the purification of the saliva samples for the small sample volumes. Carbamazepine is well absorbed from the digestive tract, and usually occurs in high concentrations in saliva. This allows for the use of a small sample volume (200 μL). Preliminary studies showed that the use of Strata X-C columns, which were rinsed with water and a mixture of water and methanol (1:1), and saliva samples diluted with 2% formic acid solution and a mixture of water and methanol (1:1), is a good procedure for purifying saliva samples [22]. Applied to the analysis of neuroleptics and carbamazepine, it allowed for good saliva purification and an analysis of patient samples. However, when the saliva contained the active metabolite carbamazepine, the extraction process was inefficient, as evidenced by the relatively low recovery [23]. Therefore, it was decided to use Phree columns, which do not require activation, and the purification process is simplified by adding to the analyzed sample a 1% formic acid solution in methanol or acetonitrile as a deproteinizing agent. If a methanol solution is used, the ratio of sample volume to solution is 1:4, and if acetonitrile is used, the ratio is 1:3. In both cases, after the addition of the deproteinizing solution, the samples were shaken for 10 min, centrifuged, and then applied to the columns. The obtained extract was evaporated and the dry residue was dissolved in the mobile phase. However, the chromatographic analysis of blank saliva samples showed that in both cases, there was no complete purification of the extracts, and the retention times of the recorded peaks coincided with those of the analytes. This makes it impossible to analyze low concentrations of carbamazepine and its metabolite in saliva, as illustrated in Figure 3a,b.

#### 3.2.2. Modification of the Deproteinization Process

**Figure 4:**
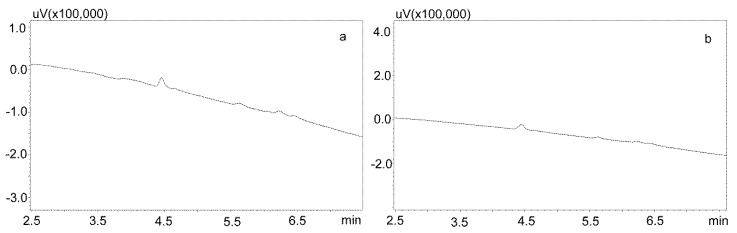
Chromatograms of blank saliva samples deproteinized with: (**a**) 1% formic acid in methanol; (**b**) 1% formic acid in acetonitrile. The separation was carried out using a Luna Omega 1.6 µm Polar C18 column and a mobile phase consisting of water with formic acid (solvent A) and acetonitrile (solvent B) at a flow of 1 mL/min.

**Figure 5:**
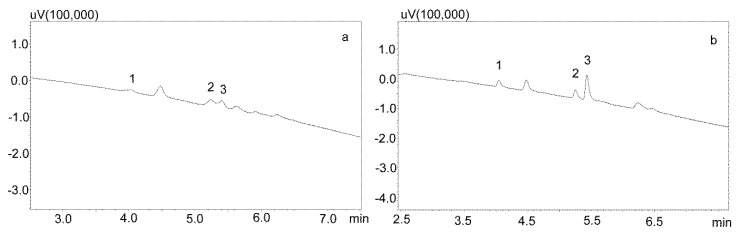
Chromatograms of saliva sample spiked with 1000 ng/mL after deproteinization with 1% formic acid in acetonitrile. The compounds were separated using a Luna Omega 1.6 µm Polar C18 column and a mobile phase consisting of water with formic acid (solvent A) and acetonitrile (solvent B) at a flow of 1 mL/min. (**a**) without evaporation; (**b**) after evaporation and dissolving in mobile phase. 1—carbamzepine-10,11 epoxide; 2—carbamazepine; 3—IS (chlordiazepoxide).

For the modification of the sample purification, deproteinization was applied using the same formic acid solutions as for the purification of saliva using the Phree columns (Section 3.2.1.). Then, 800 or 600 μL of 1% formic acid solution in methanol or acetonitrile were added to 200 μL of saliva, respectively, mixed, shaken for 10 min, centrifuged, and then 10 μL of supernatant was analyzed by UHPLC. This procedure allowed for a very good purification of blank saliva samples, as illustrated by Figure 4a,b. However, in the case of spiked samples, regardless of the deproteinization solution used, the shape of the peaks was unsatisfactory, as they were very wide at the base. This caused difficulties in determining their surface area for low concentrations of the analyzed compounds (Figure 5a).

Another modification of the purification of saliva containing carbamazepine and its active metabolite included the evaporation of a formic acid solution and the dissolution of the dry residue in the mobile phase. In addition, to reduce the use of solvents, and because of the small differences in the amount of impurities visible on the chromatograms after deproteinization, a 1% formic acid solution in acetonitrile was used. For this purpose, the supernatant was evaporated and the dry residue after dissolution in the mobile phase was centrifuged again and analyzed chromatographically (Figure 5b).

### 3.3. Method validation

The linearity specified for both analytes in the concentration range 10–5000 ng/mL has shown that the method is linear and meets the specified acceptance criterion (R^2^ ≥ 0.99, Table 2). The LOQ for both analytes was set at 10 ng/mL. The detailed test results in Table 3 also indicate that the developed method is precise. CV for inter- and intra-day precision did not exceed 15% for any of the tested concentrations.

The selectivity of the method was determined by analyzing 10 blank saliva samples obtained from 10 healthy volunteers. No interference resulting from the presence of endogenous compounds was observed in any of the tested samples, which confirms the absence of peaks with retention times similar to those of analytes.

### 3.4. Extraction and Absolute Recovery

The extraction recovery was determined for two concentrations of analytes (30 ng/mL—low QC; 3750 ng/mL—high QC), comparing the peak areas of the analytes extracted by the method developed with those of blank saliva samples loaded after deproteinization and solution evaporation. For both analytes, extraction recovery was close to 100%. A slightly lower value (>95%) was obtained for the saliva loaded with carbamazepine epoxide at 30 ng/mL.

The absolute recovery was also determined for two concentrations of analytes (30 ng/mL—low QC; 3750 ng/mL—high QC), comparing the peak areas of the extracted analytes with the peak areas obtained during the analysis of six neat standards at each concentration. The lowest absolute recovery value (>81%) was observed for 30 ng/mL, for carbamazepine and its active metabolite.

Both the extraction recovery and absolute recovery for both analytes at all of the tested concentrations met the criteria (>50%). Therefore, it should be considered that the method developed for saliva purification is suitable for the determination of carbamazepine and carbamazepine epoxide, as presented in Table 4.

### 3.5. Stability

The stability of analytes in the biological matrix and during the storage of samples in the autosampler was tested. For three concentrations (30 ng/mL—low QC; 2000 ng/mL—medium QC; 3750 ng/mL—high QC), the results obtained in Table 5 indicate that carbamazepine and carbamazepine-10,11 epoxide are stable under the tested conditions.

### 3.6. Clinical Application

**Figure 6:**
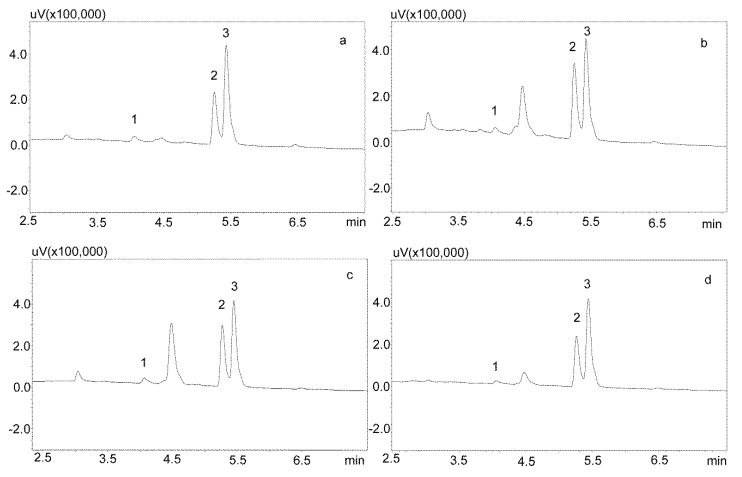
Chromatogram of saliva extracts of patients treated with carbamazepine: 1—carbamzepine-10,11 epoxide; 2—carbamazepine; 3—IS (chlordiazepoxide). (**a**) Patient 15: 569.1 ng/mL (1), 1668.1 ng/mL (2); (**b**) Patient 14: 559.1 ng/mL (1), 2456.8 ng/mL (2); (**c**) Patient 7: 664.1 ng/mL (1), 2334.2 ng/mL (2); (**d**) Patient 2: 428 ng/mL (1), 1817.8 ng/mL (2). The compounds were separated using a Luna Omega 1.6 µm Polar C18 column and a mobile phase consisting of water with formic acid (solvent A) and acetonitrile (solvent B) at a flow of 1 mL/min.

The saliva samples came from 20 patients treated with carbamazepine in mono- and poly-therapy. The method used allowed for determining the concentrations of both analytes in all of the samples. Detailed data, including age, gender, drug dose, and concentration of analytes in saliva, are presented in Table 6, while examples of patients’ saliva extract chromatograms are shown in Figure 6. The average age of the patients was 40 years. The mean concentration of carbamazepine determined in the male saliva was 1882 ng/mL, and was more than twice as high as in the female saliva (967 ng/mL). The mean metabolite concentration was similar, and was 299 ng/mL for men and and 249 ng/mL for women.

## 4. Discussion

The method developed in this study enables the determination of carbamazepine and carbamazepine-10,11 epoxide in human saliva. Although carbamazepine was introduced to the treatment as early as the 1960s, it is still one of the most frequently used substances in central nervous system diseases. It is also used in the pharmacotherapy of epilepsy in children. Carbamazepine has a strong influence on its own metabolism and the drugs used in parallel, and so it is advisable to monitor its concentration in the body.

As a result of the confirmed correlation of its concentrations in blood and saliva, various studies have been carried out to develop methods of carbamazepine determination in these diagnostic materials. The methods for the determination of anti-epileptic drugs in blood have often been modified to determine carbamazepine in saliva [2,3,6,8,9,17,18]. Most often, saliva samples were 1 mL [14,18,22,23,25] or more [3,6,8], and were rarely less than 1 mL [15,21,24,27]. The method developed in this study allows for the determination of carbamazepine and its active metabolite, in as little as 200 µL of saliva. This is particularly useful in children and those carbamazepine users who suffer from dry mouth.

The developed method is characterized by a good linearity in the concentration range of 10–5000 ng/mL (R2 > 0.999). The concentration range was selected to coincide with the range of therapeutic concentrations observed in patients’ saliva [4,6,7,8,15,16].

Purification of saliva samples involved deproteinization, which proved to be the simplest and most effective method of extraction of the analyzed analytes. In many studies, saliva is purified by liquid–liquid extraction [8,14,16,24,25,26], which provides a high recovery of analytes >89% [24,25], but requires volatile and toxic solvents such as chloroform. In turn, the use of SPE does not need toxic solvents, but carbamazepine interacts strongly with the adsorbent, which reduces its recovery [23]. In this study, the extraction recovery of the developed method exceeded 95% and the absolute recovery exceeded 81% for both analytes.

All previous literature reports confirm that the analyzed compounds are persistent and their decomposition during storage does not exceed 10% [20]. These studies also confirm the stability of the analytes under the tested conditions. The highest concentration reduction, at 6%, was recorded for 30 ng/mL carbamazepine epoxide in saliva samples stored at 8 °C.

The developed method allows for the determination of concentrations of both analytes in all of the tested patient samples. The determined concentrations ranged from 262 to 4891 ng/mL for carbamazepine, and from 120 to 664 ng/mL carbamazepine-10,11 epoxide, which is consistent with the literature data [4,6,8].

## 5. Conclusions

The aim of this study was to develop a method for saliva purification and to determine the concentration of carbamazepine and its active metabolite, carbamazepine-10,11 epoxide. Among the extraction methods studied, deproteinization with 1% formic acid solution in acetonitrile proved to be the most effective. The volume of the sample used for analysis does not exceed 200 µL. The method is linear in the range 10–5000 ng/mL (R^2^ > 0.999), and is characterized by high precision, with the highest CV of 12.82% for carbamazepine and 13.58% for carbamazepine epoxide. Moreover, the extraction efficiency exceeded 95%, and the absolute recovery exceeded 81%. Both carbamazepine and its active metabolite were stable under all of the tested conditions. Using the developed method, the concentrations of both analyzed compounds were determined in all of the saliva samples from the patients.

## Figures and Tables

**Table 1:** UHPLC (Ultra High Performance Liquid Chromatography) gradient program.

Time (min)	Gradient (Percentage of Solvent B by Volume)
0.01	10
0.5	10
10.0	55
12.0	90
12.2	10
15.0	10

**Table 2:** Calibration curves and validation parameters for the developed procedure. SD—standard deviation; LOQ—limit of quantification.

Calibration curve *y = ax + b* (*n* = 4)	Carbamazepine	Carbamazepine-10,11 epoxide
Range (ng/mL)	10–5000	10–5000
Determination Coefficient (R^2^)	0.99958 ± 0.000171	0.99975 ± 0.000006
Slope a ± SD	0.00025 ± 0.0001	0.000175 ± 0.00015
Intercept b ± SD	0.004825 ± 0.002326	0.004875 ± 0.003333
LOQ (ng/mL)	10	10

a—slope; b—intercept; SD—standard deviation; LOQ—limit of quantification.

**Table 3:** Intra- and inter-day validation parameters (*n* = 20). CV—coefficient of variation.

Analyte	Carbamazepine		Carbamazepine-10,11 epoxide			
Quality Concentration	Intra-day (% CV)	Inter-day (% CV)	Accuracy (%)	Intra-day (% CV)	Inter-day (% CV)	Accuracy (%)
Low (30 ng/mL)	6.39	12.82	95.35	6.02	13.58	109.28
Medium (2000 ng/mL)	4.97	5.32	98.51	4.18	3.14	97.59
High (3750 ng/mL)	2.69	2.67	98.65	2.67	2.77	97.93

CV—coefficient of variation.

**Table 4:** Extraction and absolute recovery of carbamazepine and its active metabolite.

Analyte	Carbamazepine		Carbamazepine-10,11 epoxide	
Concentration (ng/mL)	30	3750	30	3750
Extraction Recovery (%)	99.09	101.19	95.89	97.37
Absolute Recovery (%)	82.19	97.07	81.13	92.61

**Table 5:** Stability study of analytes in spiked saliva stored in a fridge at 8 °C and after freeze–thaw cycles at −21 °C. The stability of the extracts kept in the autosampler at 15 °C for 72 h. Values in the table are expressed as %loss.

Analyte	−21 °C			8 °C			15 °C		
	30	2000	3750	30	2000	3750	30	2000	3750
	Difference (%)								
Carbamazepine	−2.25	−2.16	−1.44	−3.91	−2.53	−1.93	−0.30	−1.12	−1.72
Carbamazepine-10,11 epoxide	−2.45	−1.92	−0.48	−6.23	−3.56	−2.75	−3.64	−2.3	0.38

**Table 6:** Patients data. Concentrations of carbamazepine and carbamazepine-10,11 epoxide found in the saliva of 20 patients male (M) and female (F) patients treated with carbamazeine.

Patient	Gender	Age (Year)	Dose (mg/day)	Carbamazepine Concentration (ng/mL)	Carbamazepine-10,11 epoxide Concentration (ng/mL)
1	M	31	800	1192.3	231.8
2	M	44	400	1817.8	428.0
3	M	50	400	4891.0	226.8
4	M	58	800	4839.8	348.5
5	M	49	700	1165.7	229.8
6	M	33	400	262.2	120.7
7	M	46	800	2334.2	664.1
8	M	44	800	344.7	122.0
9	M	55	400	690.7	168.9
10	M	51	800	1285.1	448.3
11	F	22	1200	464.0	161.1
12	F	49	400	282.2	127.5
13	F	26	600	472.9	172.9
14	F	37	1200	2456.8	559.1
15	F	31	1200	1668.1	569.1
16	F	29	1800	613.5	182.1
17	F	19	600	784.3	273.3
18	F	31	800	1119.9	152.7
19	F	69	900	607.6	147.7
20	F	30	400	1198.2	142.7
